# Antioxidant Effect of *Chrysanthemum morifolium* (Chuju) Extract on H_2_O_2_-Treated L-O2 Cells as Revealed by LC/MS-Based Metabolic Profiling

**DOI:** 10.3390/antiox11061068

**Published:** 2022-05-27

**Authors:** Ge Zhan, Men Long, Kai Shan, Chong Xie, Runqiang Yang

**Affiliations:** 1 School of Biological Science and Food Engineering, Chuzhou University, Chuzhou 239000, China; zhangeczxy@163.com (G.Z.); czxy_lm@163.com (M.L.); 2 Whole Grain Food Engineering Research Center, College of Food Science and Technology, Nanjing Agricultural University, Nanjing 210095, China; shankai@njau.edu.cn (K.S.); xiechong@njau.edu.cn (C.X.)

**Keywords:** chrysanthemum, antioxidant, L-O2 cells, metabolites

## Abstract

Chrysanthemum has a long history of being used to attenuate various oxidative stress-related discomforts and diseases; however, its mechanisms remain unclear. In this study, the antioxidant effect of chrysanthemum aqueous extract was investigated, and the potential mechanisms were explored via a metabolomics study. Chrysanthemum extract could significantly inhibit hydrogen peroxide (H_2_O_2_)-mediated cell death in L-O2 hepatocytes. Propidium iodide staining and annexin V-PI dual staining revealed that the antioxidant effect of chrysanthemum extract was related to the relief of cell cycle arrest and inhibition of non-apoptotic cell damage. The activities of antioxidant enzymes including superoxide dismutase (SOD), catalase (CAT) and glutathione peroxidase (GSH-Px) were also upregulated by chrysanthemum extract. Through metabolomics studies, it was found that chrysanthemum extract mainly targeted the arginine synthesis pathway and purine metabolism pathway, in which antioxidation-related endogenous substrates including L-arginosuccinate, citrulline and inositol monophosphate were significantly upregulated by chrysanthemum extract. These results indicated that chrysanthemum extract can antagonize oxidative stress through multiple pathways and have potential therapeutic applications.

## 1. Introduction

Natural products have a long history of use as drugs, drug precursors, and/or complementary health adjuvants due to their abundant health benefits. Among them, plants are the most important source of natural products due to their wide availability. *Chrysanthemum morifolium* (Chuju) is one of the four top cut-flowers in the world. In addition to being an ornamental plant, the flowers of chrysanthemum also show potential medicinal effects [1]. For a long time, chrysanthemum and its extracts have been used to relieve multiple discomfort symptoms in Asia, such as improving liver function and alleviating red and itchy eyes [2]. Recent studies indicated that *C. morifolium* ethanol extract attenuated lipopolysaccharide-induced acute lung injury in mice by increasing the activity of total-antioxidant capacity and decreasing the contents of malondialdehyde [3]. In addition, *Chrysanthemi* was also found to alleviate acetaminophen-induced rat liver injury via inhibiting oxidative stress and apoptosis [4]. Although numerous studies have demonstrated the antioxidant effects of chrysanthemum in vitro and in vivo and some potential antioxidant substances including caffeic acid, apigenin, luteolin as well as glucosides have been identified, the molecular mechanism is still poorly studied [5].

Isolation and identification of the bioactive molecules in a mixture is a classical strategy for studying natural products. A lot of natural compounds including alkaloids, phenolics, and terpenes in chrysanthemum have been identified as antioxidants [6]. For them, the predominant effects may involve the activation of Nrf2-related antioxidant pathways and scavenging of free radicals [7,8,9]. However, researchers found that the biological activities of natural extracts are much higher than those of single bioactive molecules due to the interaction between molecules at pharmacodynamic and pharmacokinetic levels, suggesting that the synergistic effect between multiple compounds cannot be overlooked [10]. Examples exist in which *Hypericum perforatum* (*H. perforatum*) extract-derived procyanidin B2 and hyperocide can significantly improve the bioavailability and benefits of hypericin from *H. perforatum* [11]. This indicates the significance of systematic studies on the investigation of antioxidant mechanisms of natural products.

The redox system in humans is a homeostatic network coupling with material and energy metabolism. Multiple metabolic participants such as respiratory chain, NADPH oxidase and lipoxygenase generate oxides and peroxides, while GSH and NADPH play a central role in the body’s antioxidant response as substrates of multiple enzymes [12]. This suggests that changes in overall metabolic pattern can be meaningful to identify the redox state during cellular oxidative stress. It has been found that oxidative stress may contribute to the reduced influx of L-arginine in platelets, while L-arginine could lower lipid peroxidation and maintain mitochondrial functions and neuronal survival [13,14]. Moreover, antioxidants contribute to decreased levels of creatine kinase activity in serum; creatine and its metabolite creatine phosphate can also act as antioxidants and prevent behavioral and oxidative stress alterations. Their metabolism is disrupted by redox disorders [15,16]. It follows that the profile of metabolites can accurately reflect the body’s redox levels and help reveal the involved molecular mechanisms. Simultaneously, metabolomics can provide a good scenario for the study of antioxidant mechanisms by tracking the metabolic flux of antioxidants, oxides and endogenous metabolites.

In this study, we found that chrysanthemum extract can significantly inhibit oxidative stress in L-O2 hepatocytes in a dose-dependent manner. Through metabolomic analysis, we further found that chrysanthemum extract exerted antioxidant effects by regulating the activity of antioxidant enzyme and targeting the arginine synthesis pathway and purine metabolism pathway. This study provides a new perspective for understanding the regulation of redox by plant extracts at the metabolic level, which may help to better clarify the medicinal mechanism of chrysanthemum.

## 2. Materials and Methods

### 2.1. Materials

The chrysanthemum (chuju) extract was obtained by aqueous extraction. In brief, after drying, chrysanthemum was extracted at 80 °C for 5 min by adding deionized water at 1:20 (*w*/*w*). Extractions were performed three times in total. The raw extract was pooled and filtered through a 0.22 μm filter. The filtrate was then freeze-dried. L-O2 human hepatocyte was commercially acquired from Zhong Qiao Xin Zhou Biotechnology Co., Ltd. (Shanghai, China). Hydrogen peroxide (H_2_O_2_) was purchased from Hushi (Shanghai, China). Both the 3-(4,5)-dimethylthiahiazo(-z-y1)-3,5-di-phenytetrazoliumromide (MTT) cell proliferation and cytotoxicity assay kit (M1020) and 2′,7′-dichlorofluorescin diacetate (4091-99-0) were purchased from Solarbio Science & Technology Co., Ltd. (Beijing, China). The malondialdehyde (MDA) assay kit (TBA method, A003-1-2), superoxide dismutase (SOD) assay kit (WST-1 method, A001-3-2), glutathione peroxidase (GSH-Px) assay kit (A005-1-2), catalase (CAT) assay kit (A007-1-1) and annexin V cell apoptosis assay kit (G003-1-2) were purchased from JianCheng Bioengineering Institute (Nanjing, China). The cell cycle assay kit (propidium iodide staining method) was purchased from Zhi En biology (Hefei, China). The mitochondrial membrane potential assay kit with JC-1 (C2006) was purchased from Beyotime (Shanghai, China).

### 2.2. Cell Culture

L-O2 human hepatocyte was maintained in RPMI-1640 supplemented with 10% fetal bovine serum, 100 U/mL penicillin and 100 µg/mL streptomycin. Cells were cultured at 37 °C in a humidified incubator with 5% CO_2_. To induce H_2_O_2_-mediated oxidative stress, 2 × 10^4^/well cells were plated in a 96-well plate and cultured for 24 h. After removing the medium, H_2_O_2_ at final concentrations of 0, 1.0, 1.2, 1.4, 1.6, 1.8, 2.0, 3.0, 4.0, 5.0, 6.0 and 7.0 mM in medium was added into different wells. After that, the incubation continued for 4 h. To explore the effect of chrysanthemum (chuju) extract, 2 × 10^4^/well cells were plated in a 96-well plate and cultured for 24 h. After removing the medium, chrysanthemum extract at final concentrations of 0, 50, 100, 200, 400, 800, 1600 and 2400 μg/mL was added into different wells and vitamin C (50.0 μg/mL) was also added as a positive control. After 12 h of treatment, H_2_O_2_ was added at a final concentration of 7.0 mM and cells were cultured for another 4 h.

## 3. Test Method

### 3.1. Cell Viability Assay

For each well of 96 well plates, 25 μL of 5 mg/mL MTT solution was added. After 4 h of incubation, MTT solution was removed and 150 μL per well dimethyl sulfoxide was added to dissolve formazan. The absorbance values were measured using a microplate reader at a wavelength of 490 nm. Each group contained four replicates. Cell viability = (A_intervention group_ − A_blank group_)/(A_control group_ − A_blank group_) × 100%.

### 3.2. Measurement of MDA Content and SOD, GSH Px, CAT Activities

The medium from cultured cells was removed and cells were digested using 0.25% trypsin without EDTA. Cell pellets were collected through centrifuge at 2000× *g* for 5 min. After one wash using PBS, cells were lysed with lysis buffer (150 mM NaCl, 50 mM Tris HCl, 1 mM EDTA, 1% TritonX-100, pH 7.4). After a centrifuge at 12,000× *g* for 20 min, supernatant was collected and protein concentration was determined using the BCA method. According to the instructions of the kits, the content of MDA and the activities of SOD, GSH Px and CAT were measured. The content of MDA is expressed in nmol/mg protein, and the activities of SOD, GSH Px and CAT are expressed in U/mg protein.

### 3.3. Cell Cycle Analysis

The single-cell suspension was washed using PBS, and cells were fixed by icy 70% ethanol for 2 h. After two washes, cells were incubated with 100 μL RNAse (20 μg/mL) at 37 °C, then stained with 400 μL PI solution (10 μg/mL) at 4 °C for 30 min. Then, cells were analyzed through a BD Accuri™ C6 flow cytometer. The data were processed by Flowjo software.

### 3.4. Mitochondrial Membrane Potential Analysis

After digestion, cell pellets were collected through centrifuge at 2000× *g* for 5 min. The 100 to 600 thousand cells were resuspended in 0.5 mL medium containing serum and stained according to the instruction. Then, the cells were analyzed through a BD Accuri™ C6 flow cytometer. The data were processed by Flowjo software.

### 3.5. Apoptosis Assay

The single-cell suspension was washed using PBS, and cells were resuspended in 0.5 mL binding buffer. Then, cells were stained with Annexin-V and PI for 15 min and analyzed through a BD Accuri™ C6 flow cytometer. The data were processed by Flowjo software.

### 3.6. Reactive Oxygen (ROS) Detection

Cells were stained with 15 μM 2′,7′-dichlorofluorescin diacetate (DCF) for 30 min. After washing with PBS, cells were treated with H_2_O_2_ and chrysanthemum extract. Then, the cells were digested with trypsin. After centrifugation, cell pellets were resuspended in 0.5 mL PBS and analyzed through a BD Accuri™ C6 flow cytometer. The data were processed by Flowjo software.

### 3.7. UHPLC-QE-MS Untargeted Metabolomics

Metabolites Extraction: 200 μL of water was added to the samples. After 30 s vortex, the samples were frozen and thawed with liquid nitrogen 3 times. The samples were sonicated for 10 min in an ice-water bath. Fifty microliters of homogenate was used to measure protein concentration. Then, 600 μL acetonitrile:methanol = 1:1 was added to the remaining part and transferred to a 2 mL EP tube. After 30 s vortex, the samples were sonicated for 10 min in an ice-water bath and were incubated at −40 °C for 1 h and centrifuged at 12,000 rpm for 15 min at 4 °C. The 700 μL of supernatant was transferred to an EP tube and dried in a vacuum concentrator. Then, acetonitrile:methanol:water = 2:2:1, with isotopically labelled internal standard mixture, was added in proportion. After 30 s vortex, the samples were sonicated for 10 min in an ice-water bath. Then, the samples were centrifuged at 12,000 rpm for 15 min at 4 °C. The resulting supernatant was transferred to a fresh glass vial for analysis. The quality control (QC) sample was prepared by mixing an equal aliquot of the supernatants from all of the samples.

LC-MS/MS Analysis: LC-MS/MS analyses were performed using a UHPLC system (Vanquish, Thermo Fisher Scientific, Waltham, MA, USA) with a UPLC BEH Amide column (2.1 mm × 100 mm, 1.7 μm) coupled to a Q Exactive HFX mass spectrometer (Orbitrap MS, Thermo). The mobile phase consisted of 25 mmol/L ammonium acetate and 25 ammonia hydroxide in water (pH = 9.75) (A) and acetonitrile (B). The autosampler temperature was 4 °C, and the injection volume was 3 μL. The QE HFX mass spectrometer was used for its ability to acquire MS/MS spectra on information-dependent acquisition (IDA) mode in the control of the acquisition software (Xcalibur, Thermo). In this mode, the acquisition software continuously evaluated the full scan MS spectrum. The ESI source conditions were set as follows: sheath gas flow rate as 30 Arb, Aux gas flow rate as 25 Arb, capillary temperature 350 °C, full MS resolution as 60,000, MS/MS resolution as 7500, collision energy as 10/30/60 in NCE mode, spray voltage as 3.6 kV (positive) or −3.2 kV (negative), respectively.

Data preprocessing and annotation: The raw data were converted to the mzXML format using ProteoWizard and processed with an in-house program, which was developed using R and based on XCMS, for peak detection, extraction, alignment, and integration. Then, an in-house MS2 database (BiotreeDB) was applied for metabolite annotation. The cutoff for annotation was set at 0.3.

### 3.8. Statistics

For all bar graphs, data are presented as mean ± SD. Two-tailed Student’s *t*-test was used for the statistical comparison of two groups and one-way analysis of variance (ANOVA) was used for multiple comparisons. In cases where all groups shared an identical sample size, the Tukey test was adopted. *p* < 0.05 was considered significant. Statistical differences between the groups were marked with different letters.

## 4. Results and Analysis

### 4.1. The Effect of H_2_O_2_ and Chrysanthemum Extract on Cell Viability

**Figure 1:**
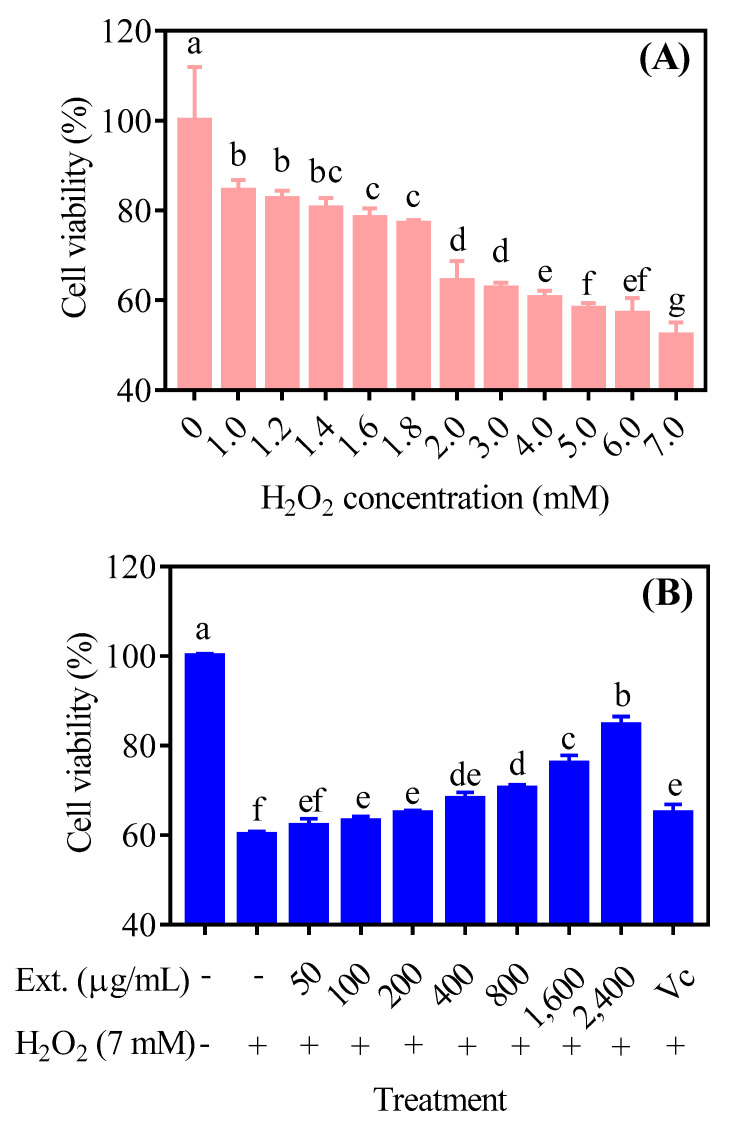
H_2_O_2_ and chrysanthemum extract on cell viability. (**A**) LO2 cells were treated using different concentrations of H_2_O_2_ for 4 h and the cell viability was determined by MTT assay. (**B**) LO2 cell was pretreated with different concentrations of chrysanthemum extract for 12 h and H_2_O_2_ for another 4 h. Cell viability was determined by MTT assay. Ext.: chrysanthemum extract, Vc: vitamin C. Three independent experiments were performed. The lower case letters indicate significant difference at *p* < 0.05 for different treatments.

Here, we employed a H_2_O_2_−induced cell death model to research the antioxidant effects of chrysanthemum extracts. Through the MTT assay, it was found that the cytotoxicity of H_2_O_2_ was significantly correlated with its concentration and 7.0 mM H_2_O_2_ can induce the strongest cell death (Figure 1A). Then, we tried to rescue the cell death with chrysanthemum extract and vitamin C (as a positive control). As expected, chrysanthemum extract attenuated H_2_O_2_-induced cell death, and its effects exhibited a concentration-dependent manner (Figure 1B). Notably, chrysanthemum extract of 800 μg/mL and above exhibited better protective abilities than vitamin C, implying the powerful antioxidant properties of chrysanthemum.

### 4.2. Protective Effect of Chrysanthemum Extract against Oxidative Damage of Cells

#### 4.2.1. Cell Cycle and Apoptosis

**Figure 2:**
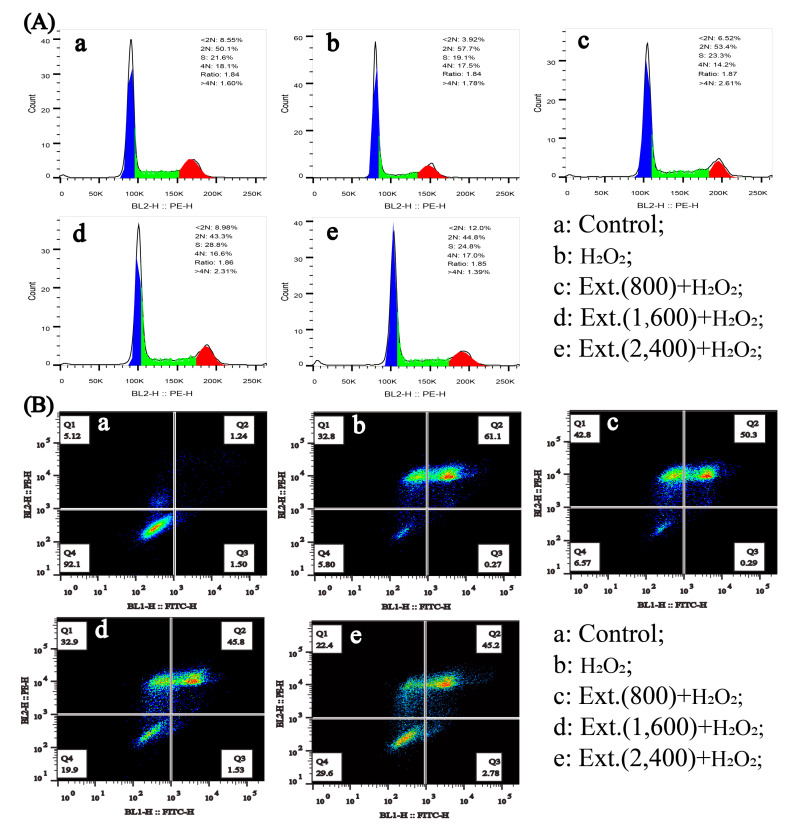
Chrysanthemum extract inhibited H_2_O_2_-induced cell cycle arrest and non-apoptotic cell death. L-O2 cell was pre-treated with different concentrations of chrysanthemum extract and treated with H_2_O_2_. (**A**) Cells were stained with PI and RNAse, then analyzed with a flow cytometer through the BL-2 channel. (**B**) Cells were stained with PI and annexin-V-FITC, then analyzed with a flow cytometer through the BL-1 and BL-2 channels. Ext.: chrysanthemum extract. Blue represents 2N (G0, G1 phase), green represents S phase, and red represents 4N (G2 phase).

H_2_O_2_ induces damage of DNA and proteins, then initiates subsequent cellular responses. Through propidium iodide (PI) staining, we found that H_2_O_2_ significantly induced the cell cycle arrest as indicated by an increased cell number in G0/G1 phase and a decreased cell number in S phase as well as G2/M phase (Figure 2A). In contrast, chrysanthemum extracts can effectively antagonize the effect of H_2_O_2_ (Figure 2A). On the other hand, H_2_O_2_ also caused severe non-apoptotic cell death, which was shown by a significant annexinV and PI double positive staining and a low annexin−V single positive staining (Figure 2B). Chrysanthemum extract can also inhibit such cell death in a concentration−dependent manner.

#### 4.2.2. MDA, ROS Content and Mitochondrial Membrane Potential

**Figure 3:**
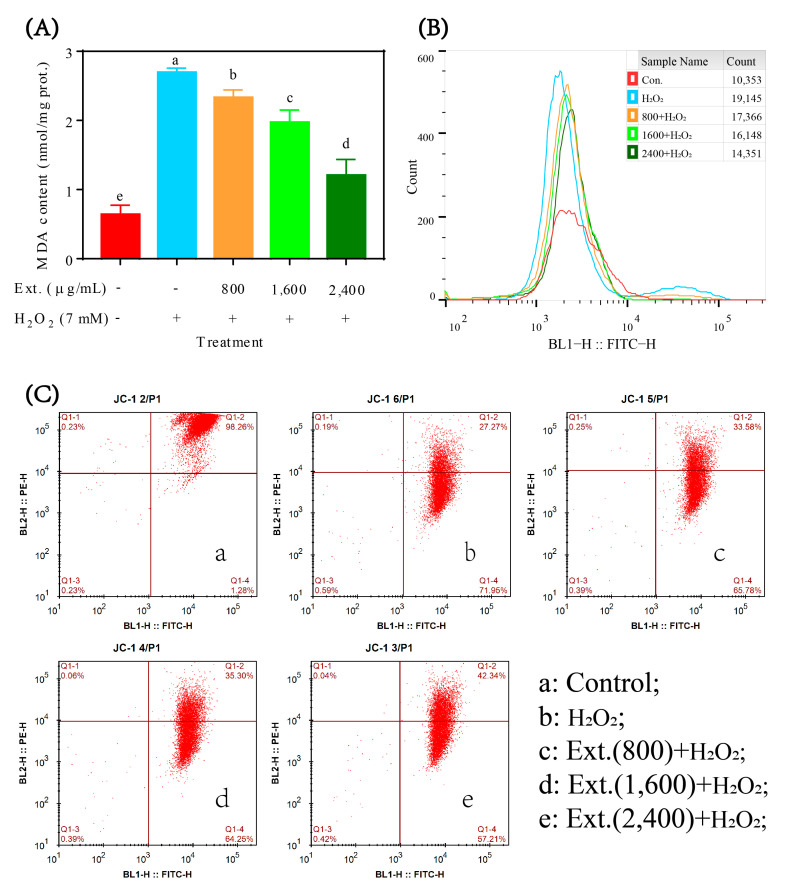
MDA, ROS content and mitochondrial membrane potential. (**A**) TBARS was performed to evaluate the lipid peroxides in L-O2 cells treated with chrysanthemum extract and H_2_O_2_. (**B**) ROS content was measured by DCF staining followed by flow cytometry. A small peak to the right of the main peak indicates a cell population containing high levels of ROS. (**C**) Mitochondrial membrane potential was measured by JC-1 staining followed by flow cytometry. FITC (BL1) indicates monomeric JC1, while red fluorescence (BL2) indicates J−aggregates. Ext.: chrysanthemum extract. The lower case letters in figure (**A**) indicate significant difference at *p* < 0.05 for different treatments.

Since the cytotoxicity of H_2_O_2_ depends on oxidative stress induction, it was speculated that the protective effect of chrysanthemum extract may come from its antioxidant effect. TBARS showed a significant lipid peroxidation induced by H_2_O_2_, while chrysanthemum extract can effectively suppress it (Figure 3A). Consistent with the changes in lipid peroxidation levels, H_2_O_2_ can also induce the boost in intracellular ROS, which was shown by a high DCF peak (Figure 3B). However, chrysanthemum extract strongly inhibited the upregulation of intracellular ROS (Figure 3B). ROS can induce a decrease in mitochondrial membrane potential and JC-1 staining can characterize such change through the decrease in J-aggregates (BL-2) (Figure 3C). Chrysanthemum extract significantly rescued the mitochondrial membrane potential dysfunction, indicating the functional effect of chrysanthemum extract (Figure 3C).

### 4.3. Antioxidant Enzyme Activities

**Figure 4:**
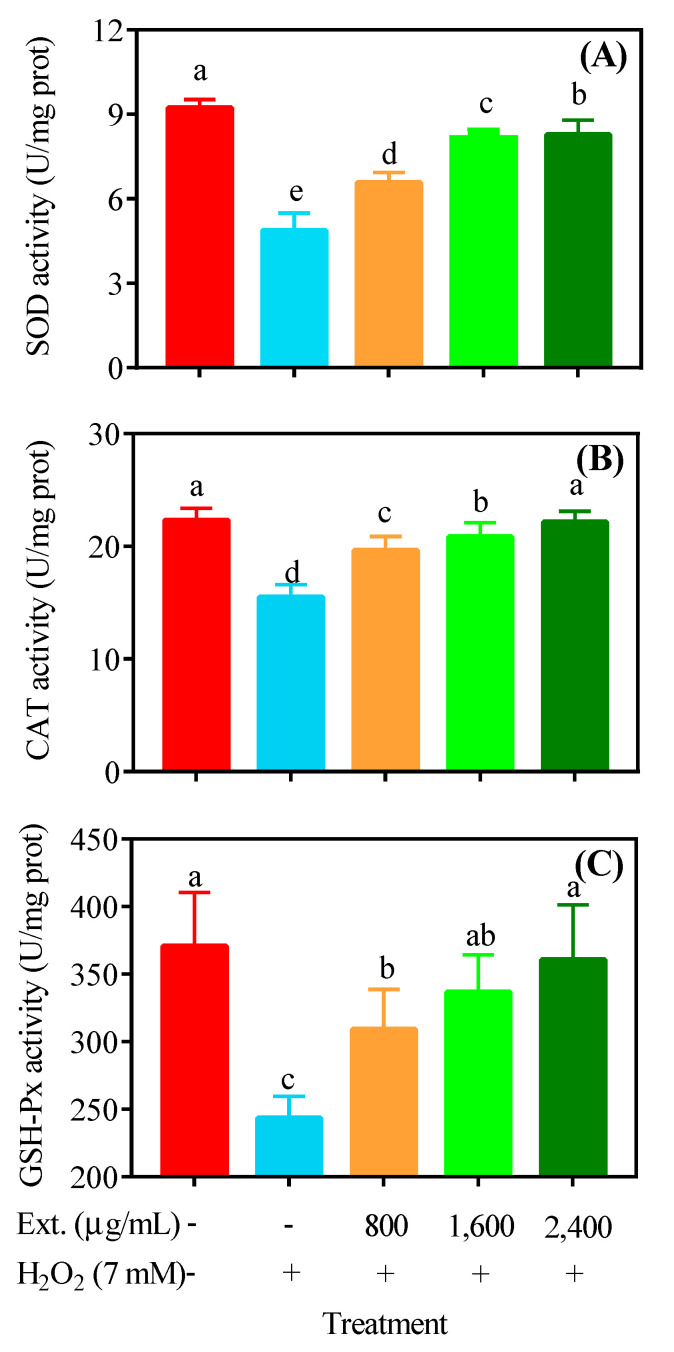
Antioxidant enzyme activities. L−O2 cells received different treatments and were lysed to evaluate the activities of SOD (**A**), CAT (**B**) and GSH−Px (**C**). Ext.: chrysanthemum extract. The lower case letters indicate significant difference at *p* < 0.05 for different treatments.

In addition to targeting oxides and peroxides directly, modulation of antioxidant enzyme activities is also a potential mechanism of action of natural products. We found that H_2_O_2_ significantly disrupted the activities of three important antioxidant enzymes—SOD, CAT and GSH−Px—while chrysanthemum extract effectively restored them (Figure 4).

### 4.4. Untargeted Metabolomics Analysis

#### 4.4.1. Principal Component Analysis

**Figure 5:**
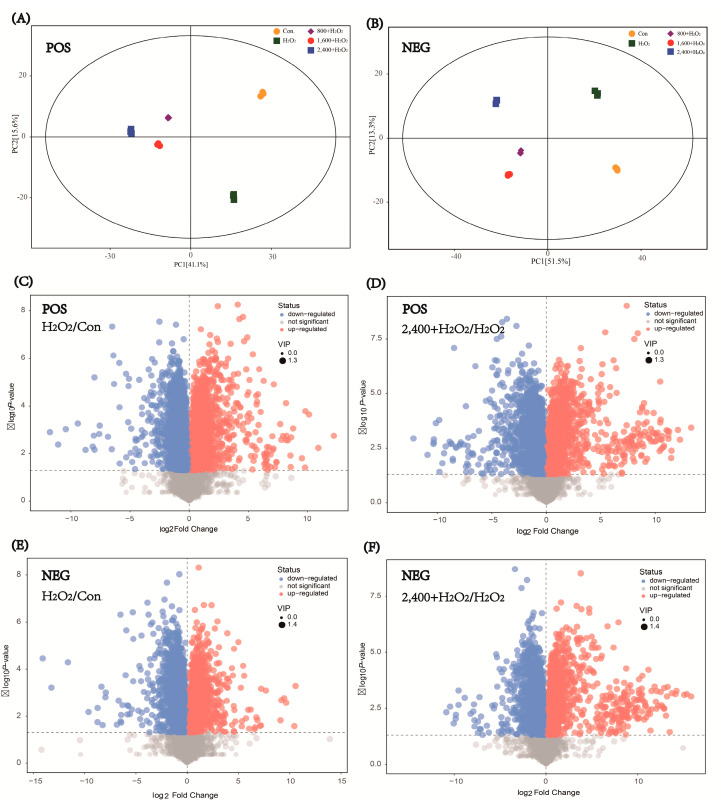
H_2_O_2_ and chrysanthemum extract induced significant metabolic changes. L−O2 cell treated with H_2_O_2_ and chrysanthemum extract were lysed for a metabonomic analysis. According to the profile of metabolites, principal component analysis was performed (**A**,**B**). The volcano plot was generated according to the differences in metabolite abundance (**C**–**F**). POS: data achieved from positive ion mode of mass spectrum; NEG: data achieved from negative ion mode of mass spectrum. Con.: control; H_2_O_2_: 7 mM H_2_O_2_; 2400 + H_2_O_2_: 2400 μg/mL chrysanthemum extract and 7 mM H_2_O_2_.

To explore the antioxidant mechanism of chrysanthemum extracts, we performed an untargeted metabolomics analysis. Whether in positive or negative ion mode, we can observe that H_2_O_2_ induced significant metabolic changes in L−O2 cells (Figure 5). Through a principal component analysis, we found that the antioxidant effect of chrysanthemum extract was accompanied by the change in cell metabolic profile (Figure 5A,B). Significantly, the metabolic profiles shaped by different concentrations of chrysanthemum extracts were similar, implying low side effects of chrysanthemum extract. Moreover, compared with the difference between the H_2_O_2_ group and the control group, chrysanthemum extract introduced more significant metabolic changes (Figure 5C–F).

#### 4.4.2. Pathway Analysis

**Figure 6:**
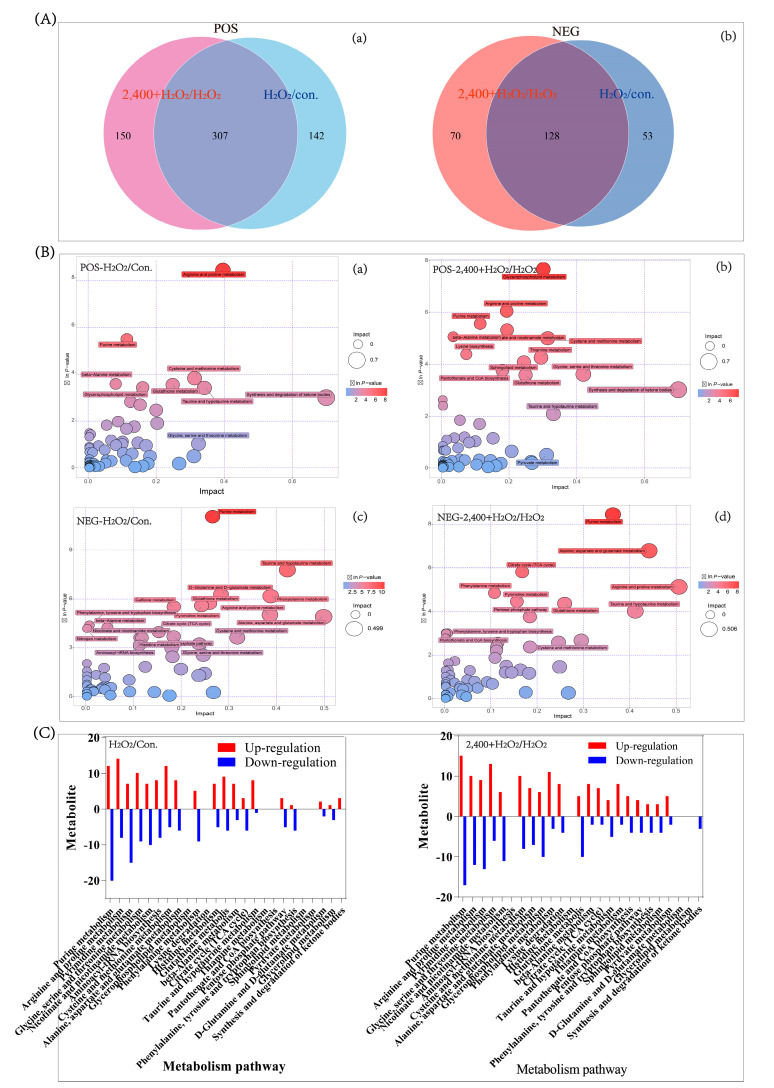
Metabolic pathway analysis. (**A**) Venn diagram showing differential metabolites of H_2_O_2_/Con. and chrysanthemum extract/H_2_O_2_. (**B**) Bubble plot showing the differential metabolic pathway enriched by metabolites. (**C**) Bar chart showing the differential metabolic pathway enriched by metabolites. POS: data achieved from positive ion mode of mass spectrum; NEG: data achieved from negative ion mode of mass spectrum. Con.: control; H_2_O_2_: 7 mM H_2_O_2_; 2400 + H_2_O_2_: 2400 μg/mL chrysanthemum extract and 7 mM H_2_O_2_. In figure (**A**), “a” represents the positive ion mode and “b” represents the negative ion mode; In figure (**B**), “a,b” represent the positive ion mode, and “c,d” represent the negative ion mode.

To evaluate the potential functions of these metabolites, we observed the quantity of metabolite that changed. The Venn plot showed that most of the metabolites influenced by H_2_O_2_ were also regulated by chrysanthemum extracts (Figure 6A). This suggested that the effects of chrysanthemum extract and H_2_O_2_ would share a similar profile of target. The main co-targets include the metabolites involved in metabolism of nucleotide bases and amino acid, especially the pathway of purine metabolism, arginine and proline metabolism (Figure 6B). Furthermore, the bar chart showed the co-targets of chrysanthemum extract with H_2_O_2_ (Figure 6C), implying the high specificity of chrysanthemum extract in antioxidation.

Further analyses found three types of metabolites: (1) chrysanthemum extract showed no effect on H_2_O_2_−treated cells including creatine and creatinine; (2) chrysanthemum extract enhanced the effect of H_2_O_2_ including L-Ornithine, phosphocreatine and D-ribose 5-phosphate; (3) chrysanthemum extract reversed the effect of H_2_O_2_, including L-citrulline, N-(L-arginino) succinate, N-acetylornithine, N-acetyl-L-glutamate and inosine monophosphate (IMP) (Table 1).

**Figure 7:**
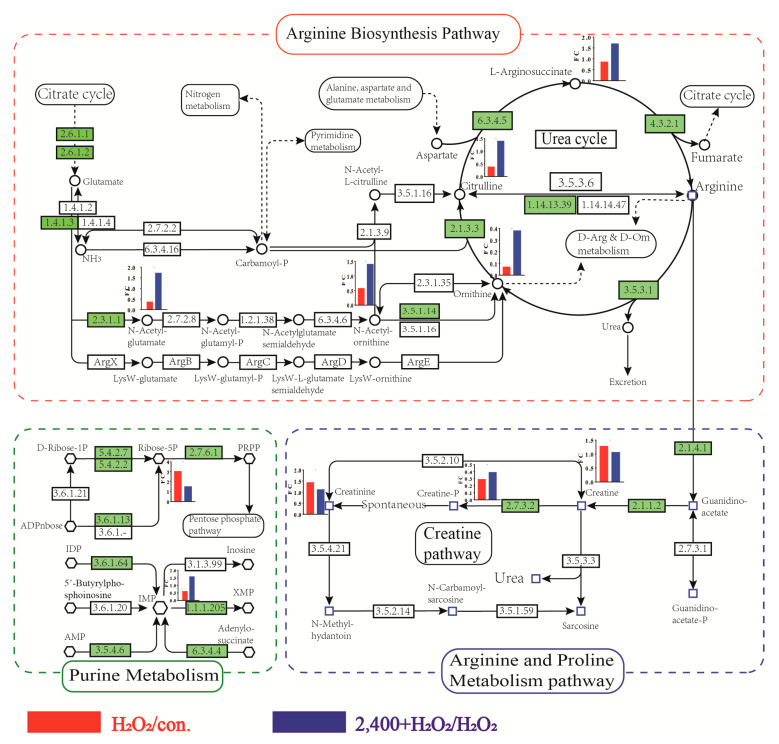
H_2_O_2_ and chrysanthemum extract regulated metabolic pathway. FC: fold change; Con.: control; H_2_O_2_: 7 mM H_2_O_2_; 2400 + H_2_O_2_: 2400 μg/mL chrysanthemum extract and 7 mM H_2_O_2_. Green represents the presence of enzymes in the human body.

Mapping metabolites into pathways helps to understand the role of H_2_O_2_ and chrysanthemum extract on cell metabolism. In the arginine biosynthesis pathway, H_2_O_2_ induced a reduced reaction chain composed of N-Acetyl-L-glutamate, N-acetylornithine, L-Ornithine, L-citrulline and N-(L-arginino) succinate, while chrysanthemum extract reversed it except L-Ornithine. Moreover, chrysanthemum extract increased IMP which is decreased by H_2_O_2_, whereas in arginine and proline metabolism, chrysanthemum extract did not influence the effect of H_2_O_2_ which is indicated by the changes in creatine and creatinine and the reduction in phosphocreatine was more severe (Figure 7). Therefore, the antioxidant effects of chrysanthemum extract may be associated with regulating the arginine biosynthesis pathway and the generation of IMP.

## 5. Discussion

Chrysanthemum extracts exhibited significant hepatoprotective effects, but the mechanism was still unclear. In this study, through in vitro experiments, it was found that chrysanthemum aqueous extract exhibited significant antioxidant functions by effectively downregulating ROS content and lipid peroxidation. Then, chrysanthemum extract further inhibited H_2_O_2_-induced decreases in mitochondrial membrane potential and hepatocyte death. H_2_O_2_ is a well-established inducer of oxidative stress damage which can induce an increase in ROS [17]. ROS can damage DNA and interfere with DNA replication, thus causing cell cycle arrest in the G1 phase [18]. Meanwhile, cell cycle arrest and damage to membrane phospholipids by ROS can further cause cell necrosis or necroptosis [19,20]. This fully indicated that the cytoprotective effects of chrysanthemum extract mainly involved its antioxidant effects targeting ROS. Meanwhile, this also explained the protective effect of chrysanthemum extract on mitochondria, as ROS is also a disruptor of mitochondrial function [21].

Physiologically, the intracellular oxides and peroxides are constantly produced, while a series of antioxidant enzymes can scavenge most of these cytotoxic substances and maintain the redox balance. SOD, CAT and GSH-Px constitute the basic antioxidant system in cells. SOD converts the highly reactive superoxide anion into relatively stable H_2_O_2_, while CAT further reduces H_2_O_2_ to water. The family of GSH-Px, i.e., GPX, contains seven reviewed members catalyzing the reduction of a wide range of substrates including H_2_O_2_ and lipid peroxides. Here, chrysanthemum extract increased the total activity of SOD, CAT and GSH-Px underlying its antioxidant mechanism. Previous works have provided some possible mechanisms to explain how antioxidant enzymes are regulated by small molecule compounds. α-Tocopherol can increase the expression of superoxide dismutase [22], pioglitazone can increase catalase activity by changing the BLC structure [23] and selenium can enable the activity of GSH-Px as a cofactor [24]. Given that, it was supposed that there might also be similar molecules in chrysanthemum extract able to regulate the expression of antioxidant enzymes or directly enhance the activity of enzymes.

Previous studies often focused on the discovery and purification of bioactive substances from natural products. Flavonoids and triterpenes are common natural compounds found in chrysanthemum in which apigenin, apigenidin and faradiol, etc., have been identified as antioxidant and anti-inflammatory factors [25,26,27,28]. This is consistent with our findings on the antioxidant effect of chrysanthemum. Despite all of this, more than the study of the action of a single substance, the synergy of bioactive substances and the joint action on multiple targets cannot be ignored. Thus, we believe that, for natural extracts with complex components, it is more meaningful to study the collection of functions by means of omics.

Through metabolomics, we found that arginine biosynthesis, arginine and proline metabolism and purine metabolism were the main targets of chrysanthemum extract. As precursors of arginine, L-arginosuccinate and citrulline were inhibited by H_2_O_2_ but upregulated by chrysanthemum extract. As L-arginine induces antioxidant response via stimulation of glutathione synthesis and activation of the Nrf2 pathway [29], we speculate that the effect of chrysanthemum extract may be related to promoting arginine synthesis and downstream metabolism. Although citrulline was upregulated, ornithine, which is the precursor of citrulline, was downregulated by chrysanthemum extract. Previous work showed that aminoacylase-1 (3.5.1.14) was increased during oxidative stress, suggesting that chrysanthemum extract might inhibit the aminoacylase-1 and therefore cause the accumulation of N-acetylornithine and further decreases in ornithine [30]. Significantly, H_2_O_2_ also decreased ornithine. However, this may be caused by a deficiency of the precursor N-acetylornithine.

Inosine monophosphate (IMP) is an essential compound for de novo nucleotide biosynthesis and metabolism of energy, proteins, and antioxidants [31]. Mitochondrial DNA is susceptible to oxidation because of the frequent exposure to ROS. Meanwhile, ATP may promote benefits to oxidative phosphorylation and help repair ROS-induced damage to mitochondrial DNA. IMP can be converted to ADP or GDP and, subsequently, to ATP or GTP, thus playing an important role in DNA repair [32]. Here, H_2_O_2_ caused the exhaustion of IMP, while chrysanthemum extract could significantly restore it, suggesting an additional antioxidation mechanism. The increased IMP guarantees the energy of cells during oxidative stress and helps the scavenging of oxides. In addition, it is also able to ensure the repair of ROS-damaged DNA and this may be the potential mechanism by which chrysanthemum extract can unblock ROS-mediated cell cycle arrest.

## 6. Conclusions

In L-O2 hepatocytes, chrysanthemum extract can effectively inhibit oxidative stress and cell death caused by H_2_O_2_. During this process, the levels of ROS and lipid peroxide decreased, the mitochondrial membrane potential recovered and the activity of antioxidant enzymes increased. Mechanistically, chrysanthemum-extract-mediated upregulation of arginine biosynthesis and IMP synthesis may contribute to the enhancement of antioxidant capacity.

## Figures and Tables

**Table 1:** Metabolites regulated by H_2_O_2_ and chrysanthemum extract.

Metabolites	No.	RT (s)	Related Pathway	H_2_O_2_/Con.		2400 + H_2_O_2_/H_2_O_2_	
				*p* Value ^a^	FC ^b^	*p* Value	FC
L-Ornithine	C00077	515.79	Arginine biosynthesis	4.59 × 10^−5^	0.07	5.16 × 10^−5^	0.38
L-Citrulline	C00327	402.96	Arginine biosynthesis	5.16 × 10^−5^	0.38	2.11 × 10^−2^	1.40
N-(L-Arginino)succinate	C03406	477.96	Arginine biosynthesis	2.22 × 10^−3^	0.86	1.55 × 10^−4^	1.69
N-Acetylornithine	C00437	403.96	Arginine biosynthesis	5.71 × 10^−4^	0.57	6.40 × 10^−3^	1.40
N-Acetyl-L-glutamate	C00624	318.93	Arginine biosynthesis	3.07 × 10^−4^	0.36	1.40 × 10^−2^	1.72
Creatine	C00300	360.33	Arginine and proline metabolism	4.37 × 10^−5^	1.28	1.40 × 10^−2^	1.06
Creatinine	C00791	174.29	Arginine and proline metabolism	6.30 × 10^−6^	1.43	1.36 × 10^−3^	1.11
Phosphocreatine	C02305	450.62	Arginine and proline metabolism	3.41 × 10^−6^	0.29	8.53 × 10^−5^	0.39
D-Ribose 5-phosphate	C00117	410.74	Purine metabolism	3.72 × 10^−4^	3.03	8.85 × 10^−3^	1.49
Inosine monophosphate (IMP)	C00130	22.17	Purine metabolism	2.25 × 10^−2^	0.60	4.80 × 10^−5^	1.58

RT: retention time; FC: fold change; Con.: control; 2400 extract: 2400 mg/mL. ^a^ The *p* value was calculated by one-way ANOVA and then by Tukey’s post hoc test for comparisons of multiple groups (after correction for multiple hypothesis testing and false discovery rate, 0.05). ^b^ The fold change (FC) of relative amounts of the drug treatment groups (GLA, gemcitabine and hydroxycamptothecin groups) compared with the normal control.

## Data Availability

Data is contained within the article.
